# Developing Stochastic Models for Spatial Inference: Bacterial Chemotaxis

**DOI:** 10.1371/journal.pone.0010464

**Published:** 2010-05-13

**Authors:** Yoon-Dong Yu, Yoonjoo Choi, Yik-Ying Teo, Andrew R. Dalby

**Affiliations:** 1 Department of Statistics, University of Oxford, Oxford, United Kingdom; 2 The Wellcome Trust Centre for Human Genetics, University of Oxford, Oxford, United Kingdom; Tel Aviv University, Israel

## Abstract

**Background:**

Biological systems are inherently inhomogeneous and spatial effects play a significant role in processes such as pattern formation. At the cellular level proteins are often localised either through static attachment or via a dynamic equilibrium. As well as spatial heterogeneity many cellular processes exhibit stochastic fluctuations and so to make inferences about the location of molecules there is a need for spatial stochastic models. A test case for spatial models has been bacterial chemotaxis which has been studied extensively as a model of signal transduction.

**Results:**

By creating specific models of a cellular system that incorporate the spatial distributions of molecules we have shown how the fit between simulated and experimental data can be used to make inferences about localisation, in the case of bacterial chemotaxis. This method allows the robust comparison of different spatial models through alternative model parameterisations.

**Conclusions:**

By using detailed statistical analysis we can reliably infer the parameters for the spatial models, and also to evaluate alternative models. The statistical methods employed in this case are particularly powerful as they reduce the need for a large number of simulation replicates. The technique is also particularly useful when only limited molecular level data is available or where molecular data is not quantitative.

## Introduction

Biological systems are by their very nature heterogeneous [1], [2]. The cell contains many different compartments and even in the “fluid” cytosol many protein molecules are localised through interactions with the cytoskeleton or with the membrane/membrane bound proteins [3]–[5]. It is also possible that molecules within cells could be localised dynamically by reaction-diffusion processes, which are known to establish spatial organisation in chemical and biological systems [6], [7].

Biological systems can also be characterised by their robustness and their ability to deal with both internal (intrinsic) and external (extrinsic) fluctuations [8], [9]. Organisms need to be resistant to fluctuations in environmental conditions so that they can maintain developmental and regulatory control, otherwise external perturbations could have serious consequences for development and for maintaining homeostasis.

In order to build more realistic models of biological processes we need to build models that incorporate stochastic and spatial effects [10]. One solution to this modelling problem has been the creation of agent based or mesocopic models [11], [12]. In these models the system components are treated as objects whose behaviour is described by a set of physical rules, while the rest of the cellular components are ignored and treated as contributing to the background in which the simulations take place. One of the weaknesses of this approach is that it is difficult to use the models for inference. Many potential models can be constructed with different sets of parameters or using variations on the physical rules used to describe the behaviour of the agents. It is not possible to construct an analytical framework that can be used to optimise the model parameters as would be the case in models that use ordinary differential equations where a likelihood method could be used [13].

For mesocopic and agent based methods it is not possible to construct a likelihood function for the parameters and so a trial and error approach has to be taken, where successive models are built with different sets of parameters that are tested against the experimental data by measuring the closeness of fit. For this approach to work two criteria have to apply;

1. A series of parameter values have to be constructed that reflect some prior knowledge of the system.
2. The output of the model has to be evaluated against the experimental data.

A further implicit requirement is that different models should be distinguishable by the criteria used for the evaluation of the different models. For ordinary differential equation models, parameter scan methods have been developed and phase plane analysis can be used to examine the dynamic regimes for different parameter values [14], [15]. In cases where parameters have not been determined experimentally phenomenological models can be constructed where the properties of the model can be determined. In the case of mesoscopic models increased realism comes at a cost of computational time and so there is a need to keep the number of simulations that need to be carried out to a minimum.

For mesoscopic models, model evaluation can be particularly difficult in cases where there are a large number of parameters or where the model is very complex and the experimental data only measures a small number of variables. In these cases the model is likely to be either over-fitted or the models will be indistinguishable unless there is a very sensitive scoring of models.

### Bacterial Chemotaxis

Bacterial Chemotaxis is the process by which bacteria sense gradients of specific chemicals, which can either act as attractants or repellents [16]–[18]. Attractants are usually sources of nutrients for the bacterium. In the case of the bacterium *Escherichia coli* the swimming behaviour of the cell is controlled by an alternating sequence of linear swimming behaviour punctuated by periods of tumbling motions. These two behaviours are controlled by the bacterial flagella motors that cause the whip like flagella to form a coordinated bundle which acts like a semi-rigid propeller, when they rotate counter-clockwise or for the bundle to break and tumbling to begin when they turn in a clockwise direction.

The rotation of the bacterial flagella motor is controlled by a signal transduction pathway that starts from the chemical receptors embedded in the cellular membrane that detect the attractant and repellent molecules. The signal is then passed through a series of intermediate species that are either localised close to the receptor array or that are freely diffusing in the cytoplasm. The key diffusive molecule is known as CheY which can be found in a phosphorylated and unphosphorylated state. The binding of the phosphorylated state CheY-P to the flagella motors increases the probability of a transition from counter-clockwise to clockwise rotation of the motor. So that a higher concentration of CheY-P in the cell promotes tumbling.

**Figure 1:**
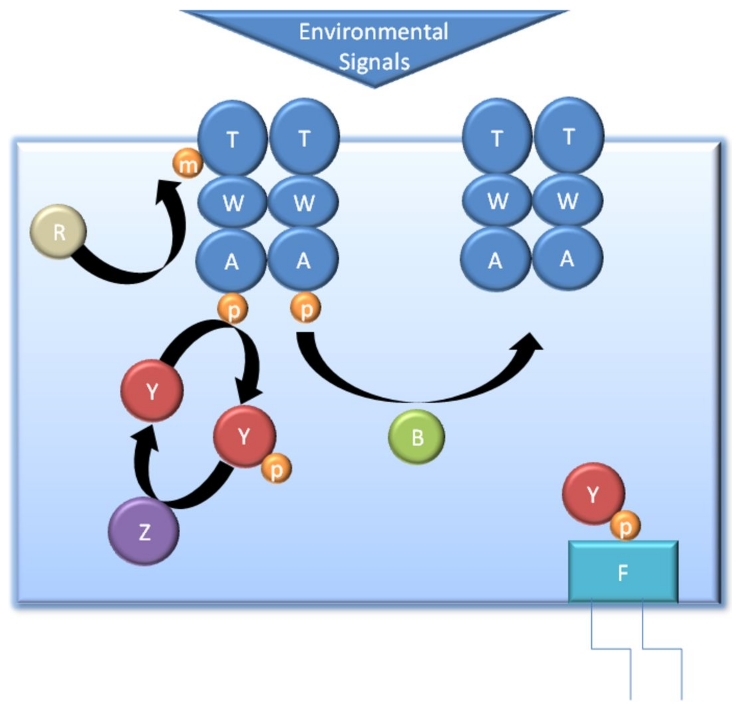
Schematic representation of the *E. coli* chemotaxis system. The Tar receptors are labelled T, CheW, CheY, CheA, CheZ, CheB and CheR are labelled as W, Y, A, B, Z and R respectively and the flagellal motor (FliM) is labelled as F. Phosphate and methyl groups are shown as orange cirles labelled p or m respectively.

Figure 1 shows a schematic representation of the chemotaxis network. In this case only one of the five types of cellular receptors is shown, this corresponds to the aspartate receptor Tar. Asparate is an example of an attractant molecule, along with sugars and most amino acids (leucine is an exception). CheY is phosphorylated by the activated form of the kinase CheA and dephosphorylated by the phosphatase CheZ. Binding of attractants to the receptor decreases the rate of CheY phosphorylation reducing the concentration of CheY-P and therefore reducing the tumbling rate.

An important feature of chemotaxis is that it detects gradients, so that in an environment where there is an equal concentration of attractant in every direction the bacterium will resume tumbling as there is no preferred direction to take. The system is very sensitive to differences in gradient and can detect differences of one molecule per cell volume per micron. Once the cell is again in a homogeneous environment it returns to its equilibrium tumbling frequency, which is the same regardless of the environmental level of attractant.

The fluctuations between counter-clockwise and clockwise rotations for the flagella motor have been measured in single cell studies for unstimulated cells, where there are no attractant or repellent molecules in the bacterial media [19]. This experimental data can be used to construct a model of the equilibrium state of the system, where we can investigate the localisation of the signal transduction pathway components. Experimental studies using green fluorescent protein labelled CheZ molecules have shown that the phosphatase is localised to the receptor array, but there is a need to independently quantify the localisation more precisely [20]–[23].

In this work we use mesocopic models of *E.coli* chemotaxis to infer the localisation of CheZ. We have developed a statistical analysis of the output of the stochastic simulations that allows the different models to be distinguished and the localisation parameters to be determined.

## Results and Discussion

The movement of a single **unstimulated** (i.e. in the absence of either repellent or attractant) bacterial cell has been recorded over 170 minutes at 0.01 second intervals. The time series has been recorded as a binary time series where −1 represents counter-clockwise rotations of the flagella motor and 1 represents clockwise rotations. The time series can be broken down into different distributions for the time intervals in clockwise and counter-clockwise motions. Korobkova and co-workers showed that the clockwise intervals are characterised by an exponential like distribution and the counter-clockwise intervals by a long-tailed deviation from the exponential distribution [19].

The stochastic simulations are based upon the experimentally determined values of the kinetic parameters for the series of chemical reactions equations that form the signal transduction network. These parameters are not part of the experimentally determined validation set and there is a non-linear relationship between them, that we are trying to determine through the simulations. The parameters that we wish to infer are the volume in which the CheZ molecules are localised (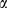) and the fraction of CheZ molecules that are localised within this volume (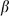). An initial simulation (Simulation 0) was run with no CheZ localisation at all, the other twelve simulations were carried out using the CheZ localisations given in table 1.

**Table 1:** CheZ localisation parameters for the simulations.

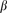	0.25	0.5	0.75	1
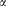				
0.01	Simulation 1	Simulation 2	Simulation 3	Simulation 4
0.05	Simulation 5	Simulation 6	Simulation 7	Simulation 8
0.1	Simulation 9	Simulation 10	Simulation 11	Simulation 12

Where 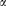 is the fraction of the cell volume around the anterior array in which the CheZ molecules are localised and 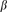 is the fraction of CheZ molecules localised in this volume.

In order to make the comparison with the experimental data the switching function from counter-clockwise to clockwise rotation of the flagella motors has to be calculated from the concentration of CheY-P molecules. This step in the reaction cannot be modelled explicitly due to the lack of experimentally determined kinetic parameters, and so this is done using a threshold transformation as described by Emonet and co-workers.
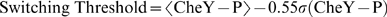
(1)


Where 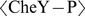 is the mean number of CheY-P molecules and 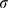
(CheY-P) is the standard deviation.

### Verifying the Equilibrium Behaviour

**Figure 2:**
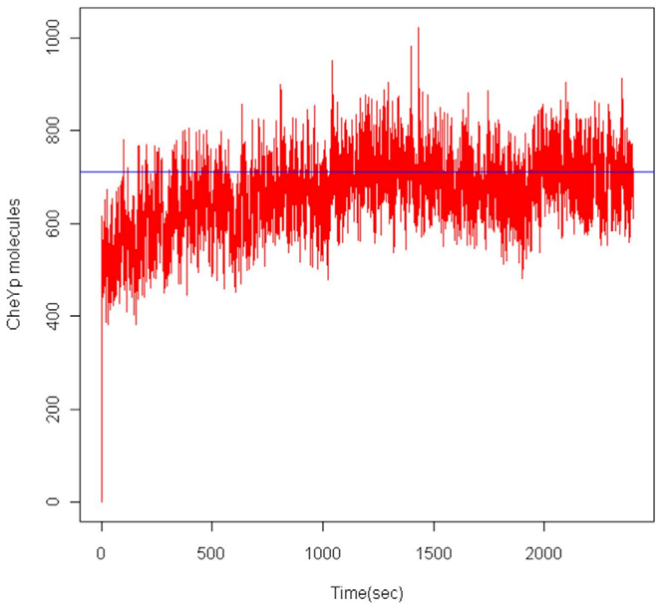
The variation in number of CheY-P molecules for the freely diffusing CheZ model.

With any stochastic simulation it is important to verify that the system is reproducing the expected variation and that the variability is not divergent. In this case the cells are unstimulated and so the signal transduction network should be at equilibrium. This implies that the variation in the levels of CheY-P should not show any significant upward or downward trend over long periods of time, but should fluctuate around a mean value. Figure 2 shows the variation in CheY-P concentration for the model where CheZ is completely delocalised. The switching threshold is shown in blue. The trace has been smoothed over a sliding window of 0.3 seconds. In this case the number of CheY-P molecules does not show any underlying linear trend and the simulation can be assumed to have reached equilibrium. This is not the case for the model where CheZ is completely localised, where there is an underlying linear trend and the system does not achieve equilibrium, and so the complete rigid localisation of CheZ is not consistent with our model.

From all of the choices of CheZ localisation parameter the simulations that did not converge to equilibrium and so were discounted from further statistical analysis were simulations 3,4,5,6 and 8.

One of the weaknesses of mesoscopic models is the computational resources that are required to carry out the calculations. As these calculations are inherently stochastic it is important to verify that the output correctly samples the possible outcomes. There are two possible approaches that have been used extensively in the molecular dynamics literature. The first is to run a single simulation for a very long time periods so that the sampling is as complete as possible and the second is to run many different short simulations and to average them [24]. In this case as the system is at equilibrium and we are measuring time series data the first, single simulation approach is more appropriate but we can verify this by checking the variability of the switching threshold from the simulations, in order to demonstrate the robustness of the method. The mean and the sample standard deviation for the switching thresholds of ten repeats of Simulation 0, where CheZ is freely diffusing are 721.8893 seconds and 0.8981 seconds respectively. This indicates that our simulator is robust and that the true mean at the 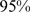 level can be found in the confidence interval between 721.2468 and 722.5317 seconds for the completely delocalised model.

### Preliminary Comparison of the Completely Localised and Freely Diffusing Models of CheZ Localisation

**Figure 3:**
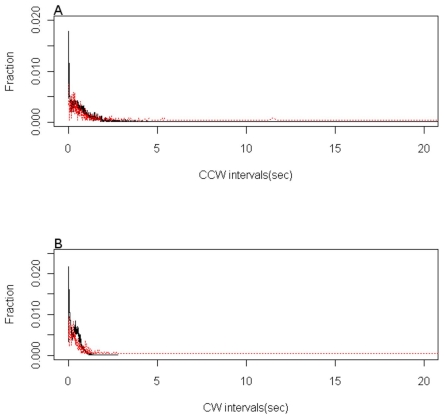
The counter-clockwise and clockwise interval distributions for the freely diffusing CheZ model.

Figure 3 shows the counter-clockwise and clockwise interval distributions for the model where CheZ is diffusing freely. In the experimental data the counter-clockwise intervals vary from 0 to 179.13 seconds, whilst that of the clockwise intervals ranges from 0 to 2.77 seconds. Previous studies have put the two sets of interval data on the same scale as counter-clockwise switching intervals of longer than 20 seconds are rare events, and so we restricted our analysis to the range 0 to 20 seconds.

An important feature of the counter-clockwise distribution is the presence of a peak in the 0 to 1.5 second region. This bimodality should arise naturally from the simulations if they are to be reliable models of the signal transduction network. Almost all of the simulations except for those with a very high degree of CheZ localisation exhibit this bimodality. A complete breakdown of the results are given in table 2. These show that our simulations can robustly reproduce the experimental data. The finding that the total localisation of CheZ cannot reproduce the empirical switching behaviour, agrees with the experimental finding that modified bacteria where CheZ cannot be localised still retain chemotactic activity [21]. Ideally we could further corroborate our model if time series data for the swimming of these modified organisms was available, so that we could check our results for the switching behaviour in the case of totally delocalised CheZ against the actual observed behaviour.

**Table 2:** Summary of the simulation results.

Simulation	Existence of the Chemical Equilibrium	Statistical Agreement	Figures
0	yes	yes	3A and 3B
1	yes	yes	5A and 5B
2	yes	yes	5C and 5D
3	no	-	-
4	no	-	-
5	no	-	-
6	no	-	-
7	yes	yes	5E and 5F
8	no	-	-
9	yes	yes	5G and 5H
10	yes	yes	5I and 5J
11	yes	yes	5K and 5L
12	yes	no	5M and 5N

The absence of a chemical equilibrium is indicated by a long term trend in the switching behaviour of the system.

The clockwise distribution only contains a single peak at 0.5 seconds. In the unlocalised CheZ simulations there is a slight shift of this peak to 0.3 seconds, but the change is much larger for the completely localised model. By considering these extreme models it indicates that complete localisation is less consistent with our model that a freely diffusive model but it is necessary to build a more detailed statistical analysis to infer the actual degree of localisation.

### Statistical Evaluation of the Simulated Interval Distributions

The simplest method for comparing an observed distribution to its underlying distribution is the use of the quantile-quantile or Q-Q plot. The Q-Q plot shows how the quantiles (percentiles for example) of the two distributions match to each other. The larger the number of quantiles that are compared the higher the resolution of the comparison. For a perfect match all of the points should lie on the diagonal. As Korobkova and co-workers pointed out in their earlier work, their will be expected variation in the switching frequency distributions of different *E.coli* cells [19]. We therefore have to consider the experimental data as a sample from the population switching distributions. We can then use this data to determine point estimators of the parameters for an appropriate population switching distribution, which will have the form that best fits the experimental data. In the case of the clockwise interval distribution this follows an exponential distribution. The Q-Q plot can therefore be used to measure the deviations between the simulations and experimental data and the exponential distribution. In the case of the counter-clockwise interval distribution there is a long tailed deviation from the exponential distribution but the underlying distribution is still exponential.

Point estimations were made using Maximum Likelihood Estimation for the rate 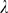 are made for both the counter-clockwise and clockwise intervals of the simulated data. For example in the freely diffusing case these are estimated to be 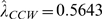 seconds and 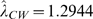 seconds.

**Figure 4:**
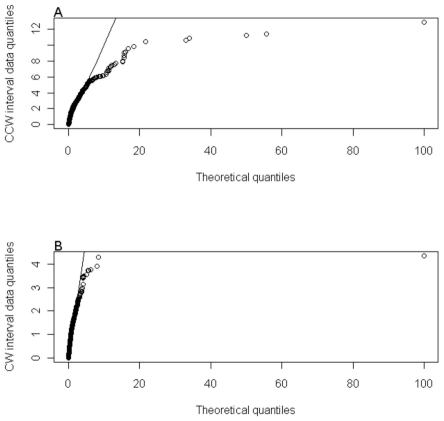
The Q-Q plot for the freely diffusing CheZ model, comparing the simulated quantiles against an exponential function fitted to the experimental data.

Figure 4 shows the Q-Q plot for the freely diffusing CheZ model comparing the quantiles for the simulated data to that of the fitted exponential exponential function. For the freely diffusing CheZ simulation the median of the counter-clockwise interval distribution is shifted 0.6 seconds. While over 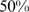 of the data points lie on the diagonal there is a significant amount of deviation for intervals longer than 5.7 seconds, which represents the long-tailed deviation from exponential. The short clockwise interval distribution is better described by an exponential function and so more of the points lie on the diagonal.

### Locally Weighted Scatterplot Smoothing

As mentioned previously there is often a lack of simulation and experimental data because of the computational cost of simulations and the resource costs of collecting the experimental data. In this case only a single empirical sampling of the swimming motions is available. By creating a smoothed distribution for the simulated and experimental data we can compare directly the different interval distributions. The smoothed distributions can be used to calculate the 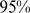 confidence intervals. If there is a complete overlap between the fitted simulated distributions and the experimentally determined confidence intervals then the two will share the same true fraction mean at the 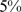 significance level. The degree of overlap will reflect the closeness of the simulation to the experimentally observed values.

**Figure 5:**
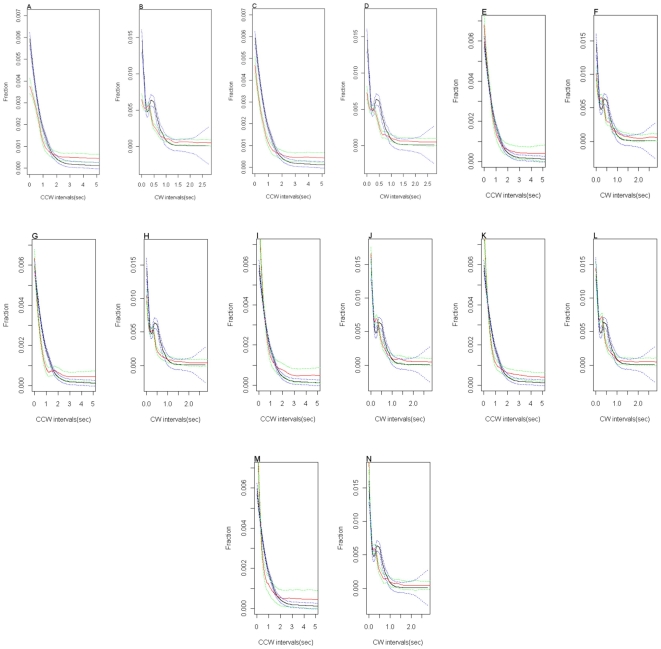
The LOESS fitting of the clockwise and counter-clockwise distributions that achieved equilibrium. The letters correspond to the simulations given in table 2.

Smoothing was carried out for the simulated and experimental data using LOESS fittings with different bandwidths, 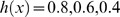 and 0.2 seconds and using different polynomial degrees such as local quadratic, cubic, linear and constant. Bandwidth and polynomial degree have to be selected carefully so as not to over-smooth the data. Figure 5 shows the LOESS fitting of the clockwise and counter-clockwise distributions from the simulations that achieved an equilibrium distribution, and the corresponding measures of statistical closeness for each simulation are given in table 3. There is excellent agreement between the two traces in many cases and the bimodality of the counter-clockwise interval distribution is also often reproduced.

**Table 3:** Statistical closeness between the simulations and the experimental data.

Simulation	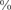 Statistical Closeness	
	CCW Intervals	CW Intervals
0	90.3	81.3
1	93.1	80.6
2	100	89.2
7	94.1	80.6
9	98.4	87.4
10	94.3	83.8
11	95.3	86

The LOESS fittings are sensitive enough to be able to distinguish between the different sets of localisation parameters. These results confirm that the absence of CheZ localisation does not diminish chemotaxis (Simulation 0 the model with freely diffusing CheZ). This result agrees with the empirical findings for cells with a truncated form of CheZ which cannot be localised. They also show that the statistically closest simulation to the experimentally observed distributions was simulation 2. In this case the amount of the CheZ localisation will be about 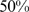 of the total number of CheZ molecules in the 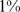 cell volume around the anterior array of the *E.coli* cell, which agrees well Manson's empirical estimation. However, we can also infer that the optimum extent of CheZ localisation at the anterior array can depend on the volume in which the CheZ is localised and that when the volume for the CheZ localisation is 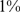 of the whole cell volume, the amount of CheZ localisation is between 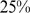 and 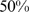 of the total number of CheZ molecules. For a 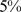 cell volume for CheZ localisation it is inferred that 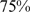 of the total CheZ molecules are present, and in the case of 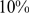 cell volume for the CheZ localisation it is between 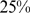 and 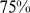 of the total number of CheZ molecules. Further simulations could be used to increase the accuracy of the inference by sampling the parameter space more finely.

### Conclusions

Using a mesoscopic model of the bacterial chemotaxis signal transduction network we have been able to infer the localisation of CheZ.

Exploratory analysis using the smoothed distribution of counter clockwise and clockwise switching intervals distinguished between parameter sets for the volume of localisation (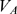) and the number of localised molecules (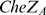), and show that incorrect values can lead to unrealistic simulations in which simulated systems do not reach the chemical equilibrium. In addition, using Q-Q plot analysis we can show that only systems that converge to equilibrium also capture the same underlying statistical distributions as that of the experimental data.

Through statistical comparisons of the chosen simulations and the experimental data, we have shown that chosen simulations from with our new algorithm agree closely with the empirically determined mean swimming responses of an observed wild-type cell in a medium in which no attractant was present. The statistically closest simulation to the experimental data was when 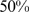 of the CheZ molecules were localised in 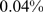 of the cell volume around the receptor arrays. Considering the counter clockwise intervals from 0s to 20 seconds, about 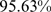 of the true fraction means of the simulated data are close to the experimental data. In the case of the clockwise intervals from 0s to 2.77 seconds, about 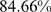 of the true fraction means of two populations are statistically close.

This is in very good agreement with the experimentally estimated degree of CheZ localisation from green fluorescent protein labelled experiments (M. Manson personal communication).

## Materials and Methods

### Simulation Algorithm

**Figure 6:**
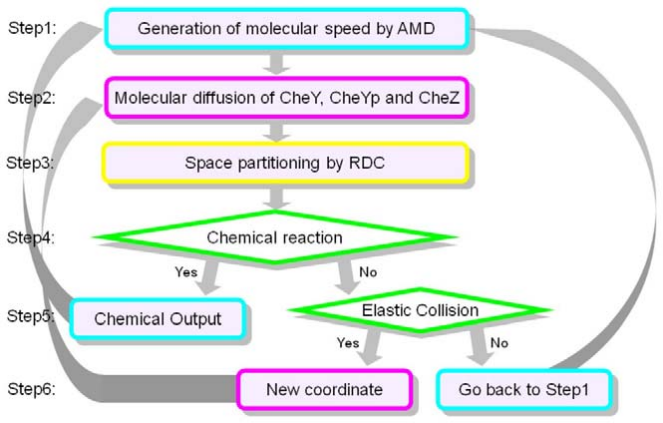
An outline of the MESMAX algorithm.

Simulations were carried out with our own implementation of the Andrews and Bray version of the Smoluchowski algorithm written in Python. Figure 6 shows the overall outline of the algorithm. The Andrews and Bray approach allows longer time-steps to be used than in the original algorithm by using effective molecular radii that indicate the volume swept out by the diffusive process in the longer time-step. These modified radii are particularly important in calculating if reactions have taken place.

The differences between this algorithm and that used within Smoldyn are the use of a Maxwell-Boltzmann distribution for the sampling of the molecular velocities, and the use of Recursive Dimensional Clustering (RDC) to simplify the collision detection process [25]. RDC uses a space partitioning algorithm to divide the simulation into volumes that contain a smaller number of objects which are then checked for collisions rather than trying the brute force collision detection for the entire simulation space.

There are a variety of possible boundary conditions that can be applied. In this case a mixture of periodic boundary conditions and impermeable surfaces was used. If during the course of a time-step the molecules make contact with the surface then it is reflected like light from a mirror by an inert impermeable surface. Any molecules that diffuse past a boundary are transferred across the system following a straight line path over the course of the time-step.

The time-step for the calculations was set to be 0.1 milliseconds as a compromise in giving the highest degree of accuracy without increasing the computational over-head unnecessarily.

### Simulation Set-up

Phosphate is much smaller than the other molecules in the simulation and so it can be treated as a dimensionless point particle. The masses of CheA, CheY (and CheY-P), CheZ and FliM are 71, 14, 24 and 38 kDA respectively. Only CheY, CheY-P and CheZ are mobile in the cytoplasm and so their diffusions constants are needed for the simulation. CheY and CheY-P have a diffusion constant of 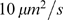 and CheZ has a diffusion constant of 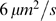 [3]. The radii of CheA, CheY (and CheY-P), CheZ and FliM were estimated to be 5.5nm, 2.3nm, 3.9nm and 2.7nm respectively using the PSA program within JOY [26].

**Figure 7:**
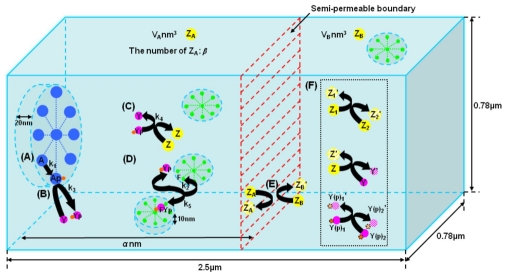
The *E. coli* simulation system.

Figure 7 shows the *E.coli* simulation system. An *E.coli* cell is approximately cylindrical with a length of 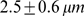 and a diameter of 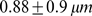 [27]. There can be a considerable difference in the ratio between length and diameter depending on growth conditions. For the simulations this is approximated to a rectangular box with a length of 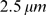 and a width and height of 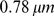. The 6700 CheA molecular dimers are located in a radial array of area 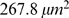, 20nm from the inside surface of the anterior cell wall. This corresponds to the active portion of the receptor-CheA complex that is responsible for signal transduction from the membrane receptors. Four rings each 45nm in diameter and made of 34 regularly spaced FliM monomers are placed on the long side walls of the cell, positioned 10nm from the inside surface. Each ring is placed randomly on a different side wall [28]. The 1600 CheZ dimers are located according to the localisation parameters either in a volume close to the anterior ray or freely diffusing in the cytoplasm [29]. The cytoplasm is then seeded with 8200 freely diffusing CheY monomers [29].

The set of reaction equations are given below and the rate constants are given in table 4.

**Table 4:** Table of kinetic rate constants.

Rate Constant	Value	Reference
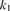	0.4 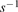	[32]
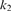	20 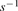	[33]
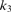	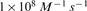	[34]
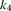	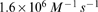	[29]
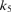	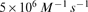	[33]

The literature values for the rate constants used in the reaction equations.

There are two unimolecular chemical reactions:
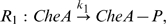
(2)

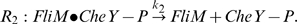
(3)


There are three bimolecular reactions:
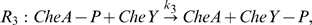
(4)

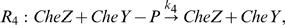
(5)

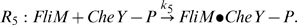
(6)


### Statistical Methods

We employed the use of exploratory procedures to examine the underlying structure between the experimental and simulated data. Since reactions within a system will eventually reach a chemical equilibrium, we assess its existence within our simulations by assessing constancy of variance and amplitude of the smoothed stochastic time evolutions of CheY-P molecules with a sliding average of width 0.3s. Subsequently we verify that our simulation results have the same underlying statistical features as that of the experimental data using a comparison of the percentiles of the theoretical and simulated distributions using a quantile-quantile (Q-Q) plot. Theoretical distributions for the counter-clockwise and clockwise interval data are generated from appropriate exponential distributions with rate parameters estimated using the maximum likelihood approach within the R statistical environment [30].

We estimate the true mean patterns of the experimental and simulated data by the use of locally weighted scatterplot smoothing (LOESS) using the loess function in R [30], [31]. LOESS fittings were carried out at different bandwidths, h(x) = 0.8, 0.6, 0.4 and 0.2, and varying polynomial degrees such as local quadratic, cubic, linear and constant for both the simulations and the experimental data. Here, the bandwidth and the polynomial degree are chosen to give the best fit to the data so as not to over-smooth it.

By applying LOESS in order to compare the simulated and experimental cases, we can compute approximate 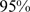 confidence intervals for the LOESS fits for each dataset. If these confidence intervals contain all of the LOESS estimates for the experimental data, this will mean that the simulation and the experimental data statistically have the same true fraction mean at significance level a = 0.05. However, if it does not contain all the LOESS estimates of the experimental data, we can still say that true fraction means of two populations are statistically close without loss of generality so long as their approximate confidence intervals overlap. In our paper, the degree of the statistical closeness between two populations is represented by the overlapped proportion of their approximate confidence intervals. This gives a more quantitative measure of the degree of agreement between the two distributions.
